# Dynamic Impact of Technology and Finance on Green Technology Innovation Efficiency: Empirical Evidence from China’s Provinces

**DOI:** 10.3390/ijerph19084764

**Published:** 2022-04-14

**Authors:** Yang Liu, Yanlin Yang, Shuang Zheng, Lei Xiao, Hongjie Gao, Hechen Lu

**Affiliations:** 1 Economics and Management School, Wuhan University, Wuhan 430072, China; 2020201050118@whu.edu.cn (S.Z.); 2020201050121@whu.edu.cn (L.X.); 2 Center for Economic Development Research and Center of Population, Resource & Environmental Economics Research, Wuhan University, Wuhan 430072, China; 00006652@whu.edu.cn; 3 School of Computer Science, Zhuhai College of Science and Technology, Zhuhai 519040, China; ghj19950920@163.com; 4 School of Management, University of Science and Technology of China, Hefei 230026, China

**Keywords:** technology and finance, GTIE, dynamic impact, super-SBM-DEA, PVAR

## Abstract

In the new stage of global economic development, we hope to achieve both economic development and environmental improvement through green technology innovation. How to effectively obtain the support of technology and finance to green technology innovation is an issue worth studying. This paper constructed an improved super-SBM-DEA efficiency measurement model and combined it with the window analysis method to measure the green technology innovation efficiency (GTIE) of Chinese provinces from 2006 to 2018. Then, based on the PVAR model, the impulse response function and Monte Carlo simulation were used to study the dynamic impact of various variables of technology and finance on GTIE. Finally, the variance decomposition was used to explore the contribution degree of each variable of technology and finance to improving GTIE. The results revealed the following: (1) the average value of China’s provincial GTIE from 2006 to 2018 was 0.42, which is relatively low and shows a trend of volatility and rising. (2) From the impulse response results, it could be seen that various variables of technology and finance have always had a positive impact on GTIE. However, there are differences in the influence degree, shock effect, and dynamic transmission mechanism. (3) The results of the variance decomposition showed that government financial technology investment had the highest contribution to the improvement of GTIE, followed by bank technology credit, then by enterprise independent R&D investment, and finally venture capital. This paper offered a reference to developing countries with regard to improving their GTIE and studying the role of technology and finance.

## 1. Introduction

With the increasing problems of population growth, resource shortage, and environmental constraints, China’s economy has gradually exposed the extensive development characteristics of “high input, high consumption, high emission, high pollution, uncoordinated, low efficiency and difficult circulation” in the process of development. This kind of extensive economic growth at the expense of resource consumption and environmental pollution is no longer sustainable [1]. Technology innovation provides a new guidance for the realization of industrial transformation and upgrading, the transformation of production modes, and the improvement of production efficiency. Technology innovation is an inevitable choice to achieve high-quality economic development in the new development paradigm with domestic circulation being the mainstay and the internal and global circulations reinforcing each other. At the same time, green development is also an important factor in high-quality development. In order to promote green development, the Chinese government has prioritized the construction of ecological civilization and proposed to build a market-oriented green technology innovation system. Under the background of the slowdown of the current world economic growth and the prominent resource and environmental constraints, as the combination of the two development concepts of “innovation-driven” and “green development”, green technology innovation can become an effective way to solve the contradiction between economic development and environmental protection [2,3].

China is a good sample for our study. In 2021, China’s social research and experimental development (R&D) investment reached 2.79 trillion yuan (RMB), an increase of 14.2% over the previous year, and the growth rate was 4% faster than the previous year. The ratio of R&D expenditure to GDP reached a new high level, close to the average level of OECD countries (data from the National Bureau of Statistics of China). According to the Global Innovation Index (GII) released by the World Intellectual Property Organization in 2021, China’s technology innovation capability ranked 12th among 132 economies, an increase of two places over the previous year, ranking first among middle-income economies. Since 2013, China’s ranking has maintained a sustained and steady upward trend, with an increase of 23 places in nine years. In recent years, the Chinese government has attached great importance to improving the ecological environment, actively controlling desertification and making positive progress in the construction of ecological civilization. Although China’s carbon emissions have remained high, showing a fluctuating upward trend (from Statista database), and China’s coal mining and imports remain the highest in the world according to the data of International Energy Agency, carbon emission intensity is declining with China’s economic growth. Carbon intensity refers to the carbon dioxide emissions per unit of gross domestic product (GDP). By the end of 2019, China’s carbon intensity had decreased by about 48.1% compared with 2005, and non-fossil energy accounted for 15.3% of primary energy consumption (the data comes from China National Bureau of Statistics). China’s external commitment to the carbon emission reduction target by 2020 has been completed ahead of schedule. As income increases, people require higher standards of environmental regulation, prompting progress in environmental protection technology [4]. In terms of finance, in 2020, China’s social financing scale increased by 34.86 trillion yuan, and financial lending to the real economy increased by 20.03 trillion yuan (data from the People’s Bank of China). China’s financial indicators are running in line with expectations, the financial system is running smoothly, the support for the real economy has increased. There is further integration of finance and the real economy, thus enhancing the industrial support of technology and finance (technology and finance refers to a systematic and innovative arrangement of a series of financial tools, financial systems, financial policies, and financial services to promote the development of science and technology, the transformation of achievements, and the development of high-tech industries, and it is an important part of the national science and technology innovation system and financial system [5]).

The main contributions of this paper were the establishment of an analytical framework for the impact of technology and finance on GTIE and exploration of the dynamic impact and contribution degree of each variable of technology and finance to GTIE. In the empirical analysis, this paper made the following designs: (1) GTIE was used to represent the development level of China’s green economy. An improved super-SBM-DEA efficiency measurement model was used and combined with the window analysis method to measure the GTIE. Among them, the super-SBM-DEA efficiency measurement model was used to consider environmental pollution factors and solve the problem that the efficiency values of multiple decision units are one at the same time and cannot be compared. Due to the different production fronts of each decision-making unit in every period, the efficiency values calculated by the super-SBM-DEA model were not dynamically comparable. Combined with the window analysis method, the dynamic incomparability problem of GTIE was solved. (2) Based on the PVAR model, the impulse response function and Monte Carlo simulation were used to obtain the dynamic impact of various variables of technology and finance on the GTIE. The variance decomposition was used to measure the contribution degree of various variables of technology and finance to the GTIE. (3) This paper compared the differential dynamic impact and contribution degree of various variables of technology and finance (government financial technology investment, enterprise independent R&D investment, bank technology credit, and venture capital) on the GTIE.

## 2. Literature Review

The concept of green technology innovation was first proposed by Fussler and James in 1996 [6]. Green technology innovation can achieve the common sustainable goals of economic development and environmental protection by reducing pollutant emissions and improving energy resource utilization efficiency, and pursue green production to achieve the co-increase of economic, social, and environmental benefits [7]. As the main way to reduce pollutant emissions, green technology innovation has always attracted the attention of scholars (Nikzad and Sedigh, 2017; Du and Li, 2019) [8,9]. Various studies have investigated the concept, driving factors, and decisive factors of green technology innovation (Aldieri et al., 2019; Li et al., 2019; Abbas and Sagsan, 2019) [10,11,12]. Existing research has proposed various GTIE measurement approaches. Generally, current research on GTIE measurement mainly uses data envelopment analysis (DEA) and the stochastic frontier analysis (SFA) model [2]. Taking into account the requirements of green environmental protection, low carbon, and energy saving, Cao and Yu (2015) improved the stochastic frontier model combined with the projection pursuit model, and calculated the GTIE of Chinese provinces [13]. Luo and Liang (2016) used the DEA method to evaluate the GTIE of Chinese industrial enterprises [14]. Fu et al. (2020) used the SFA method to measure the GTIE of manufacturing companies to discuss whether regional innovation capabilities can promote the improvement of GTIE [15]. Fang et al. (2020) used the DDF-DEA three-stage efficiency evaluation model to measure the GTIE of China’s heavy polluting industries and explored the impact mechanism of external factors [16]. Zhao et al. (2021) used the SBM model, considering unexpected output to measure the GTIE, and introduced a spatial econometric model into the convergence analyses to explore the regional differences in GTIE [17]. Zhang et al. (2021) analyzed GTIE evolution based on a network epsilon-based Measure (EBM) model and analyzed the impacts of environmental regulations [18]. Zhang et al. (2022) used a combination of the super-SBM model and the Dagum Gini coefficient method and analyzed the factors influencing industrial GTIE by constructing a spatial econometric model [19].

Finance injects funds into research and development activities, achievement transformation and industrialization of green technology innovation, also provides financing channels, which is the key factor to ensure the sustainability of green technology innovation. Technology and finance are not always unrelated and parallel, they are mutually integrated and influenced. If technology is the primary productive force for economic development, then finance is an important lever and engine for improving productivity [20]. The profit-seeking nature of financial capital and the high return of technological innovation have promoted a high degree of integration between the two. The interaction between technology and finance has been continuously enhanced, and it has gradually merged into a new research field—technology and finance. In recent years, technology and finance has developed rapidly. With the support of a series of relevant policies, theoretical research has been deepened and practical innovations have continued. Technology and finance has become an important engine of innovation-driven development, and has made important contributions to the development of the innovation economy.

Technology and finance is an emerging research field, and a mature theoretical system has not yet been formed. Foreign scholars have conducted research in this field. Schumpeter (1912) pointed out that finance can promote the generation of innovation and batches of major innovations depend on financial capital, and proved the positive impact of financial factors such as money and credit on innovation. He also emphasized the importance of financial intermediaries to the independent innovation of enterprises [21]. Hicks (1969) discussed the impact of financial market efficiency on technological innovation and believed that it was more important to popularize technological achievements through financial means than technological innovation itself, and pointed out that the industrial revolution can only happen after the financial revolution [22]. By constructing an endogenous growth model, King and Levine (1993) showed that the financial system can promote innovation by evaluating and screening potential projects, raising funds, assessing the risks of innovation activities, and estimating future cash flows [23]. Perez (2002) revealed the important role of financial capital in technological innovation from the perspective of financial capital supporting the development of technological revolution, promoting the spread of technological revolution, and giving birth to the new generation of technological revolution [24]. Hyytinena and Toivanen (2005) pointed out that the government can alleviate the financing constraints of enterprises’ technological innovation by adopting public policies, thereby promoting the development of technological innovation [25]. Benfratello et al. (2008) found that the credit input of local banks can significantly improve the success rate of technological innovation activities of enterprises in the region [26]. Neff (2012) pointed out that without financial support, it would be difficult for enterprises to carry out technological innovation [27]. The studies of Manaswi et al. (2018), Glabiszewski and Zastempowsk (2018), and Hofmann et al. (2018) all show that the integration of technology and finance can effectively improve the efficiency of resource allocation, thereby promoting the development of technological innovation [28,29,30].

In view of the importance of technology and finance to innovation, in the context of China’s implementation of an innovative country construction, technology and finance has attracted more and more domestic scholars’ attention. Fang (2010–2015) pointed out that the essence of technology and finance is the high coupling of financial system innovation and technological innovation, and further pointed out that technology and finance aims to cultivate high value-added industries, create high-paying jobs, and enhance the overall competitiveness of the economy, and it is an integral part of innovative economics [31,32]. Zhang and Zhao (2015) empirically analyzed the impact of China’s technology and finance on technological innovation and concluded that technology and finance has a significant positive effect on technological innovation in the short term. Among them, the government’s financial technology investment, enterprises’ own funds, and social capital can promote the development of technological innovation [33]. Du et al. (2017) found that technology and finance can significantly improve the regional science and technological innovation ability, and there is a spatial spillover effect [34]. Zheng and Zhang (2018) found that there is a U-shaped relationship between technology and finance and technological innovation. Only when the development of technology and finance exceeds the threshold value can it promote technology innovation [35]. Gu and Wang (2018) pointed out that technology and finance promotes technology innovation by assessing entrepreneurial ability and selecting the best innovative projects, transferring science research funds, preventing and resolving project risks, evaluating project benefits, and supervising project execution [36]. Jie (2020) took China’s “promoting the integration of technology and finance” pilot as a quasi-natural experiment, and found that promoting the integration of technology and finance significantly improved the level of innovation in the pilot areas [37]. Lv and Wang (2020) discussed the impact of technology and finance on the regional GTIE from the perspective of human capital [38].

The above literature had important reference significance for the further development of this paper, but there were also the following problems to be studied: First, in the existing research, when discussing the influence of technology and finance on the technology innovation efficiency, the DEA benchmark model was generally used in the selection of the measurement model of technological innovation efficiency, in which the measured efficiency values cannot be compared when they are both 1 and the efficiency values are not dynamically comparable. Furthermore, the “environmental pollution” factor has not been taken into account in the calculation of technology innovation efficiency. China is currently facing relatively serious environmental problems. There is an imbalance between economic development and ecological environment. The improvement of technology innovation efficiency helps to control or reduce “environmental pollution”. Therefore, it is necessary to take into account the “environmental pollution” factor when measuring technology innovation efficiency. Second, existing research has only explored the linear or nonlinear relationship between technology finance and technology innovation through static panel regression, and rarely considered the dynamic impact. Third, there are many existing studies on the supporting role of technology and finance investment in technology innovation, but there are few studies on the differential impact of different technology and finance investment on technology innovation.

In view of this, this paper expanded the literature in the following two aspects: First, a super-SBM-DEA model considering “environmental pollution” was used and combined with the window analysis method to measure the GTIE of Chinese provinces, which is helpful to measure the China’s provincial GTIE more accurately. Second, based on the PVAR model, the impulse response function and variance decomposition were used to study the dynamic impact of technology and finance on GTIE, and the influence degree, shock effect, and the dynamic transmission mechanism of each component of technology and finance on GTIE were discussed and compared, which is of great significance for the focused development of technology and finance, the promotion of GTIE, and the implementation of national innovation-driven strategy.

## 3. Theoretical Analysis

Technology and finance is a complete financial system that includes government support, bank technology credit, venture capital, and enterprise-independent R&D investment [39]. The impact mechanism of technology and finance on the GTIE is mainly manifested in the following four aspects:

(1) Technology and finance promotes the improvement of the GTIE by providing financial support for green technology innovation. The large investment amount, long cycle, slow profitability, high risk, positive externality, and non-excludability of green technology innovation activities reduce the company’s revenue expectation and cause the company’s R&D investment to be lower than the social optimal level. The intervention of technology and finance can effectively alleviate this problem. The government provides financial support for green technology innovation activities through technology policies and financial subsidies. Banks invest idle funds of individuals and households in the field of green technology innovation through technology credit. Venture capital invests funds in green technology innovation projects in the form of equity investment. Enterprises obtain financial support to compensate for the external loss of R&D activities and reduce the cost of R&D investment, thereby stimulating the enthusiasm of enterprises to engage in green technology innovation activities.
(2) Technology and finance reduces the profitability risk and liquidity risk of enterprises engaged in green technology innovation activities through the risk diversification mechanism and the liquidity creation function of the financial system, stimulates the enthusiasm of enterprises to engage in green technology innovation activities, and promotes the improvement of the GTIE. Enterprises engaged in green technology innovation have higher risks than general manufacturing or service enterprises. After investing a large amount of R&D funds, enterprises may not be able to reap the expected innovation results. Technology and finance can establish a risk diversification mechanism by providing diversified capital sources and diversified portfolio strategies to solve the problem of profitability risk effectively. At the same time, technology innovation is accompanied by high liquidity risks. The liquidity creation function of the financial system is conducive to the formation of long-term capital, and can provide stable capital flow for green technology innovation, thereby solving the problem of liquidity risk.
(3) Technology and finance guides the flow of financial resources to high-quality green technology innovation projects through the value discovery function and improves the efficiency of financial resource allocation to promote green technology innovation. There are a large number of science and technology enterprises in China, and there is a problem of information asymmetry between enterprises and investors, which can lead to adverse selection; that is, enterprises with strong innovation ability and great development potential are not be favored by funds, while some enterprises with poor innovation ability but good packaging find it easy to obtain financial support, and this mismatch of financial resources can cause economic losses. The screening and review mechanism of technology and finance for enterprises effectively solves the problem of information asymmetry between investors and enterprises so as to guide financial resources to high-quality enterprises and promote the improvement of enterprises’ GTIE.
(4) Based on the theory of signal transmission, technology and finance means endorsing the development potential of enterprises and attracting social capital to participate in green technology innovation activities. Government and other technology and finance entities provide financial support for enterprises’ green technology innovation activities, which can release positive signals to the outside. Enterprises with financial support can attract social capital investment, alleviate the financing difficulties of enterprises’ green technology innovation, and improve the GTIE.

## 4. Materials and Methods

### 4.1. Data Source and Sample Selection

The sample data for this paper came from the “China Science and Technology Statistical Yearbook”, “China Statistical Yearbook”, “China Environmental Statistical Yearbook”, “China Financial Yearbook”, “China Venture Capital Development Report”, and Zero2IPO Research Center. This paper selected the data of 30 provinces (autonomous regions and municipalities) (due to lack of data, the research samples did not include China’s Tibet Autonomous Region, Taiwan Province, Hong Kong, and Macao Special Administrative Regions) in China from 2006 to 2018 as research samples. For partially missing data, linear interpolation was used to supplement.

#### 4.1.1. Indicators Related to GTIE Measurement

Using the following indicators of innovation input, expected output, and unexpected output, the super-SBM-DEA efficiency measurement model and the window analysis method were used to calculate the GTIE of Chinese provinces from 2006 to 2018. The innovation input index adopted the full-time equivalent of R&D personnel and the R&D capital stock, the innovation expected output index adopted the technology market turnover and new product sales revenue, and the undesired output index adopted the industrial wastewater discharge and industrial waste gas discharge. The setting and data processing of each index are described as follows:

The input indicators of innovation activities were considered from two aspects of human capital input and capital input. The human capital input was represented by the full-time equivalent of R&D personnel, and the full-time equivalent of R&D personnel referred to the R&D personnel calculated by the workload. Capital investment was measured by the internal expenditure of R&D funds. In view of R&D expenditure being a flow indicator, its impact on innovation output was not only the current R&D expenditure but also the result of the accumulation of previous R&D expenditure. Therefore, the perpetual inventory method was used to estimate the R&D capital stock.

The expected output indicators of innovation activities mainly took into account the output of knowledge technology and new products. Regarding the output of knowledge technology, most of the existing literature uses the number of patent applications accepted or the number of patent applications granted. However, Griliches (1990) and Kou and Zhou (2012) believe that based on the protection of trade secrets, many innovative activities have not applied for patents, and the economic benefits brought by patents are quite different [40,41]. Therefore, the turnover of technology market was selected to approximately represent the output of knowledge technology. The technology market turnover reflects the scale of technology transactions in a region, which can effectively reflect the market value of the technology itself. For the output of new products, from the perspective of the transformation of science and technology achievements, the indicator of new products sales revenue was selected to reflect the results of innovation activities in a region. For the unexpected output index of innovation activities, from the perspective of “environmental pollution”, industrial wastewater discharge and industrial waste gas discharge were selected to represent it. (Considering that most of the solid waste has been disposed and utilized in recent years and the amount of dumping and discarding has been greatly reduced, only the discharge of industrial wastewater and industrial waste gas were selected as the unexpected output indicators.)

#### 4.1.2. Technology and Finance Indicators

At present, the academic community has not yet formed a unified indicator system to evaluate technology and finance. Based on the transmission mechanism analysis of technology and finance on GTIE, this paper drew on the studies of Zhang and Zhao (2015) [33], Huang and Li (2017) [39], and Zhang and Zhang (2018) [42], constructing the indicator system of technology and finance from four aspects: government financial technology investment, enterprise independent R&D investment, bank technology credit, and venture capital investment. The indicators are described as follows:

(1) Government financial technology investment

The government plays an important role in the practice of technology and finance in China. It not only formulates science and technology development plans and rules and regulations for supervision but also participates in financial transaction activities. Science and technology innovation activities have the characteristics of large initial investment, high sunk cost, strong uncertainty, high risk, and positive externalities, and their spillover effects can lead to market failure. Government funding for science and technology supports and guides science and technology enterprises at the beginning of their establishment or when the market fails. The government financial technology investment index was measured by the proportion of government financial technology investment to total funds raising for science and technology, which is denoted as *gov.*

(2) Enterprise independent R&D investment

Enterprise independent R&D investment refers to the funds invested by enterprises in technological innovation projects or their own funds at the initial stage of the establishment of science and technology enterprises. In China, enterprises are the main subject of technological innovation, and enterprise technological innovation has become an important part of China’s national innovation system [43]. Enterprises are not only participants in technological innovation activities but also transformers of technological innovation achievements. Enterprises increase their own investment in innovation and establish the main position of innovation, which helps to improve the overall level of technological innovation of China. The enterprise independent R&D investment index was reflected by the proportion of the independent R&D investment of the enterprise to total funds raising for science and technology, which is denoted as *com*.

(3) Bank technology credit

A large part of the funds for enterprises to engage in technological innovation activities comes from the financial market. In the financial market, indirect financing represented by banks is the main channel for enterprises to obtain funds. In addition, innovation is risky and technological innovation activities of enterprises may not necessarily be successful. There is also the risk of failure. Banks optimize capital allocation by providing enterprises with different financial capital combinations, thereby reducing the risk of engaging in technological innovation activities for enterprises. The bank technology credit indicator was represented by the proportion of bank technology credit to total funds raised for science and technology, which is denoted as *fin*.

(4) Venture capital investment

Entrepreneurial venture capital intervenes when the enterprises are starting out and need funds urgently, opening up effective financing channels for science and technology enterprises in the early stage. At the same time, it gives professional advantages to participate in enterprises’ management and decision making, and provides management assistance to help enterprises develop. It helps enterprises improve their operating efficiency and promote the professional management and use of technology and finance resources by means of “financing + intelligence”. Venture capital investment indicator was measured by the proportion of venture capital investment to total funds raising for science and technology, which is denoted as *vc*. Descriptive statistics of variables are shown in Table 1.

### 4.2. Research Methods

#### 4.2.1. GTIE Measurement Model

At present, most of the research on the measurement of GTIE adopts the DEA method. DEA uses linear programming methods to evaluate the relative effectiveness of comparable units. In actual research of efficiency evaluations, scholars mostly use the DEA method, avoiding the estimation and inference of the specific form of the production function [44]. The DEA can deal with multi-inputs and multi-outputs, and especially, the unexpected outputs and can be used to measure the GTIE considering the unexpected output of environmental pollution [45]. The classic DEA models are radial and angular measurements, which cannot fully consider the slackness of input and output and cannot accurately measure when unexpected output exists, resulting in a large deviation in the measurement results of the efficiency value. To solve this problem, Tone (2001) proposed a non-radial non-angular SBM-DEA model based on slack variables [46]. The SBM-DEA model is suitable for measuring efficiency when inputs and outputs may vary in a disproportionate manner [47]. The SBM-DEA model can directly deal with input surplus and output shortage. In the SBM-DEA model, the data unit is invariant, and each input and output slack variable can be increased uniformly to make up for the deficiencies of other models [48]. However, the SBM-DEA model still does not solve the problem that the efficiency values of multiple decision units are 1 at the same time and cannot be compared. Based on this, Tone (2002) further proposed the super-SBM-DEA model [49]. The super-SBM-DEA model combines super-efficiency and the SBM-DEA model. The basic idea of the super-efficiency evaluation method is to remove the effective evaluation unit from the set and re-evaluate. In this way, the original ineffective value evaluation remains unchanged, and the original effective value evaluation can be greater than 1, then it can be compared [48]. Suppose the production system has *n* decision-making units, and each decision-making unit has three vectors: input X, expected output $Y^{g}$ and unexpected output $Y^{b}$, and their elements can be expressed as $x\in R^{m}$, $y^{g}\in R^{s_{1}}$, and $y^{b}\in R^{s_{2}}$. Define matrices X, $Y^{g}$, $Y^{b}$ as follows: $X=\left[x_{1},\ldots ,x_{n}\right]\in R_{}^{m\times n}$, $Y^{g}=\left[y_{1}^{g},\ldots ,y_{n}^{g}\right]\in R^{S_{1}\times n}$, $Y^{b}=\left[y_{1}^{b},\ldots ,y_{n}^{b}\right]\in R_{}^{S_{2}\times n}$, where $x_{i}>0$, $y_{i}^{{}^{g}}>0$, $y_{i}^{b}>0\left(i=1,2,\ldots ,n\right)$. The super-SBM-DEA efficiency measurement model can be written as:

(1) $$\delta^{*}=\min\frac{\frac{1}{m}\sum_{i=1}^{m}\frac{\bar{x}}{x_{ik}}}{\frac{1}{s_{1}+s_{2}}(\sum_{r=1}^{s_{1}}\frac{{\bar{y}}_{r}^{g}}{y_{rk}^{g}}+\sum_{l=1}^{s_{2}}\frac{{\bar{y}}_{l}^{b}}{y_{lk}^{b}})}\;s.t.\left\{\begin{array}{l} \bar{x}\geq \sum_{j=1,\neq k}^{n}\lambda_{j}x_{j}\\ {\bar{y}}_{}^{g}\leq \sum_{j=1,\neq k}^{n}\lambda_{j}y_{j}^{g}\\ {\bar{y}}_{}^{b}\geq \sum_{j=1,\neq k}^{n}\lambda_{j}y_{j}^{b}\\ \bar{x}\geq x_{k},{\bar{y}}^{g}\leq y_{k}^{g},{\bar{y}}^{b}\geq y_{k}^{b},{\bar{y}}^{g}\geq 0,\lambda_{j}\geq 0 \end{array}\right.$$

where $\delta^{*}$ represents the super-efficiency value, $\bar{x},{\bar{y}}^{g},{\bar{y}}^{b}$ represent the slack of input, expected output, and undesired output, respectively, and λ is the weight vector. Due to the different production fronts of each decision-making unit every year, the efficiency values calculated by the super-SBM-DEA model are not dynamically comparable. The combination of the super-SBM-DEA model and the window analysis method can solve this problem very well. The window analysis method was proposed by Charnes et al. (1985) [50]. The basic idea is that the same decision-making unit in different window periods is regarded as different decision-making units. By measuring the efficiency by moving average method, it can not only calculate the efficiency of different decision-making units on the same section but also calculate the change trend of the efficiency values of all decision-making units with time, so that the measured efficiency values are dynamically comparable.

#### 4.2.2. PVAR Model

Considering that the traditional linear regression method cannot analyze the dynamic relationship between variables, based on PVAR model, this paper used impulse response function and variance decomposition to analyze the dynamic influence of technology and finance on GTIE. The PVAR model was first proposed by Holtz-Eakin et al. (1988) [51]. In this model, all variables in the system are regarded as endogenous variables, and the lag terms of all variables are considered, so there is no contemporaneous correlation problem. The estimation error caused by the endogeneity problem is avoided to a certain extent. The PVAR model can better capture the influence of the individual differences of the sample units on the model parameters [52]. The PVAR model can measure the dynamic impact of the random disturbance shock of a specific variable on each variable in the system through the impulse response function. The impulse response was realized by Monte Carlo simulation.

The Monte Carlo method was developed in the 1940s by John von Neumann, Stanislaw Ulam, and Nicholas Metropolis while they were working on the Manhattan Project at the Los Alamos National Laboratory [53]. The model is named after a gambling city in Monaco, due to the chance and random encounters faced in gambling. The Monte Carlo method presents a class of computational algorithms that rely on repeated random sampling to approximate some unknown quantities [53]. The core of the Monte Carlo method is a uniform random number generator. It is a procedure that produces an infinite stream $U_{1}$, $U_{2}$, … of $random^{a}$ numbers on the interval (0,1) [54]. Through Monte Carlo simulation, the impulse response graph of each variable to itself and other variables in the model after being shocked by one standard deviation can be obtained, which can show the dynamic influence among the variables in the model. In addition, it explores the contribution degree of various variables in technology and finance to the GTIE through variance decomposition. The PVAR model is established as follows.

(2) $$y_{i,t}=\beta_{0}+\sum_{j=1}^{p}\beta_{j}y_{i,t-j}+f_{i}+e_{i}+\varepsilon_{i,t}$$

In Formula (2), *i* represents the cross-section individual, *t* represents the time, $y_{i,t}$ is the k-dimensional endogenous variable column vector, $\beta_{0}$ is the intercept term vector, *p* represents the model lagged order, $\beta_{j}$ is the parameter matrix of the lagged variable, $f_{i}$ is the individual effect column vector, $e_{i}$ is the time effect column vector, and $\varepsilon_{i,t}$ is the random disturbance term. Since the model has individual effects that do not change with time and the explanatory variables contain the lagged terms of the explained variables, it is a dynamic panel data model with fixed effects. Therefore, we first used the intra-group mean difference method to remove the time effect, then used the forward mean difference method proposed by Arellano and Bover (1995) [55] to remove the fixed effect.

## 5. Results

### 5.1. Measurement Results of GTIE

Taking 2006–2018 as the research period, and using the above indicators of innovation input, expected output, and unexpected output and the super-SBM-DEA efficiency measurement model combined with the window analysis method, the GTIE of Chinese province was calculated. The results are shown in Table 2. From 2006 to 2018, the average value of China’s provincial GTIE was 0.42, which was at a relatively low level. However, the average value of China’s provincial GTIE increased from 0.34 in 2006 to 0.55 in 2018, showing an upward trend of fluctuation during the study period. This was evidenced by the rapid upward trend of the China Innovation Index in the Global Innovation Index (GII). In 2006, the Chinese government put forward the strategy of independent innovation and building an innovative country, and promulgated the ‘’Outline of National Medium and Long-term Science and Technology Development Plan (2006–2020)’’, which marked that the reform of China’s science and technology system has been developing towards a systematic reform. For more than ten years, through the synergy of technology innovation, management innovation, and institutional innovation, China’s independent innovation capability has improved, and the core competitiveness of the industry has enhanced. China has made remarkable achievements in building an innovative country. At the same time, building an ecological civilization is a millennium plan for the sustainable development of the Chinese nation. In recent years, China’s green development has also achieved positive results.

### 5.2. The Dynamic Impact of Technology and Finance on GTIE

#### 5.2.1. Panel Unit Root Test and Determination of Lag Order

The precondition for the establishment of the PVAR model is that the panel data is stationary. First, the panel data should be tested for stationarity, so as to avoid the pseudo-regression problem in the estimation of non-stationary variables. In this paper, the LLC test in the same root test and the ADF-Fisher test in the different root test were comprehensively adopted. The test results are shown in Table 3. The results showed that all variables in the model passed the stationarity test, and the PVAR model could be established. For the selection of the lag order, through AIC, BIC, and HQIC criteria and considering the limitation of freedom degree, the lag order was determined to be first order. This paper used the pvar2 program improved by Lian (2009) [56] on the basis of the pvar program written by the Love and Zicchino (2006) [57] for empirical analysis.

#### 5.2.2. Impulse Response Analysis

**Figure 1:**
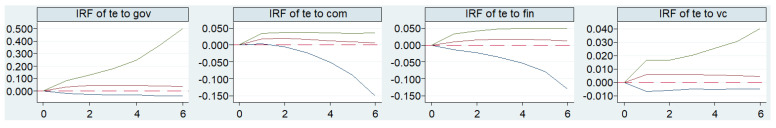
Impulse response of GTIE faced with the shock of technology and finance.

Impulse response function (IRF) can analyze the dynamic effect of a specific variable on itself and other variables after being shocked by one standard deviation while keeping other variables in the model unchanged. Through Monte Carlo simulation 500 times, a response figure of GTIE faced with one standard deviation shock of government financial technology investment, enterprise independent R&D investment, bank technology credit, and venture capital investment was made, as shown in Figure 1. The horizontal axis is the number of impulse response periods, which was set to six periods. The vertical axis represents the response degree of GTIE faced with the impact of government financial technology investment, enterprise independent R&D investment, bank technology credit, and venture capital investment. In Figure 1, the upper and lower lines are the upper and lower bounds of the 95% confidence interval, and the middle curves are the impulse response trace.

From the impulse response results in Table 4, it can be seen that after the government financial technology investment was impacted by one standard deviation, the GTIE gradually increased, reaching the peak response intensity (0.0466) in the third period, then the response degree gradually declined to zero. The direction of the response was always positive, and the cumulative effect was 0.2428. Among the variables of technology and finance, GTIE had the strongest impulse response intensity and the largest cumulative effect faced with the shock of government financial technology investment, indicating that the government made a great contribution to the improvement of GTIE during the study period. The government is a special participant in the technology and finance system. It has multiple identities. It is both a supplier and an intermediary agency. It is also the promoter and regulator of the technology and finance market. In the early stage of establishment, science and technology enterprises often encounter difficulties in financing. Due to the high risks and uncertainties, the government’s financial support has become more and more important. Since green technology innovation has a spillover effect, it can reduce the future earnings of enterprises’ R&D investment. The government’s subsidy to science and technology enterprises not only reduces the R&D investment cost of enterprises but also makes up for the loss of innovation spillovers, thereby stimulating the enthusiasm of enterprises to engage in green technology innovation activities. At the same time, the government’s increased investment in technology innovation can release positive signals to the outside world, guide the flow of social capital, attract social capital to participate in technological innovation activities, and effectively alleviate the problem of insufficient investment in innovation funds. In addition, the government’s financial technology investment can play a leverage role, and a small amount of capital investment can leverage huge social capital to participate in the technology innovation activities of science and technology enterprises [41]. Therefore, increasing government financial technology investment can effectively improve GTIE in China’s provinces.

After being shocked by one standard deviation, the enterprise independent R&D investment led to a gradual increase in the GTIE, reaching a peak response intensity (0.0208) in the second period, which ranked second among the variables of technology and finance. After that, the response degree gradually decreased and converged to zero, the response direction was always positive, and the cumulative effect was 0.0856, indicating that the increase in technology innovation capital investment by enterprises promoted the GTIE in China’s provinces. Enterprises are the direct implementers of technological innovation activities. They follow the market mechanism to allocate innovative resources, create knowledge, and obtain benefits. They are both participants in technological innovation activities and transformers of technological innovation achievements. They are the most active factor and the most important component in participating in innovation practice. Compared with other technology and finance entities, enterprises have a clearer understanding of the future development direction of the industry and the market. They can more accurately grasp innovative projects with development potential, and also grasp more market supply and demand information. The operation efficiency and management level of the enterprise are relatively high, and the enterprise itself increases investment in technological innovation and establishes the main position of innovation, which is conducive to the enterprise to take the initiative in the fierce market competition and maintain a long-term competitive advantage. At the same time, it optimizes the allocation of technological innovation resources, thus improving the GTIE in Chinese provinces.

After being shocked by one standard deviation, the bank technology credit caused the GTIE of China’s provinces to gradually increase, and reached peak response intensity (0.0177) in the third period, then the response degree gradually declined and converged to zero. The response direction was always positive and the cumulative effect was 0.0901, which ranked second in the variables of technology and finance. After being impacted by one standard deviation, venture capital investment caused a rapid increase in the GTIE, and reached peak response intensity (0.0061) in the second period, after which the response degree gradually decreased and converged to zero, the response direction was always positive, and the cumulative effect was 0.0323. It shows that during the research period, bank technology credit and venture capital investment promoted the GTIE in China’s provinces, which can be explained from the perspective of financial markets exerting their own functions. First, the financial market has the function of resource allocation, which can provide science and technology enterprises with the financial support required for their development at various stages. For example, technology-based small and medium-sized enterprises in the early stage of growth often have problems of lack of funds and high development risks, while technology-based enterprises in the mature stage also face financing difficulties for technological iterative upgrades, stable operations, and market expansion. A sound financial market system can provide targeted financial support for different types of science and technology enterprises, and promoting the integration of technology and finance is of great significance to solving financing difficulties. Second, the financial market has the function of risk sharing, which can provide enterprises with different financial capital portfolios to diversify the potential risks of technological innovation. Technology innovation has the characteristics of high cost, high risk and strong uncertainty, and requires a large amount of capital investment. By incorporating bank technology credit and venture capital into the technology and finance service system, it can provide financing support for enterprises with different risk types. It can reduce the risks faced by science and technology enterprises in the R&D process and also diversify the capital risks of investors. Third, the financial market has the function of value discovery, which can timely reflect the science and technology achievements and development prospects of science and technology enterprises, effectively guide funds to enter the most valuable innovative projects, thereby improving the efficiency of resource allocation and promoting the GTIE of Chinese provinces.

#### 5.2.3. Variance Decomposition Analysis

In order to more clearly describe and measure the difference in the influence degree of government financial technology investment, enterprise independent R&D investment, bank technology credit, and venture capital investment on the GTIE in Chinese provinces, variance decomposition was carried out on the basis of impulse response function. Variance decomposition can measure the explanatory power of each endogenous variable to the variation of all endogenous variables and can systematically analyze the contribution of each shock to the fluctuation of endogenous variables. Through 500 Monte Carlo simulations, the contribution degree of government financial technology investment, enterprise independent R&D investment, bank technology credit, and venture capital investment to GTIE in the 10th, 15th, 20th, 25th, and 30th periods were obtained, respectively, and the results are shown in Table 5.

From Table 5, it can be seen that after the 10th forecast period, the impact of various variables of technology and finance on the GTIE stabilized. In addition, it was found that the GTIE in Chinese provinces had a high degree of autocorrelation, indicating that the improvement of GTIE was not achieved overnight but was formed through long-term development and accumulation. The contribution degree of government financial technology investment, enterprise independent R&D investment, bank technology credit, and venture capital investment to GTIE was stable at 0.301, 0.032, 0.040, and 0.005, respectively.

During the study period, the government’s financial technology investment contributed the most to the GTIE. China’s current financial system is not perfect, and the government’s support and guidance for science and technology innovation activities is particularly important. During the research period, the contribution degree of enterprise independent R&D investment to the GTIE ranked third, and the lack of motivation for enterprises’ independent R&D investment was due to both their own reasons and external environmental constraints. For small- and medium-sized enterprises, survival is the first priority. The cycle of technology R&D is long and the risk is high. They are more inclined to invest limited funds in other areas with quick results. For large enterprises, there is a lack of effective incentive mechanisms, and China’s current laws and regulations on the protection of independent intellectual property rights are not perfect, resulting in a lack of motivation for enterprises to invest in independent research and development. The contribution degree of bank technology credit to GTIE ranked second. In China’s existing financial system, indirect financing is still dominant, and bank credit is an important source of funding for technological innovation [58], which was better than venture capital in improving the GTIE. The contribution degree of venture capital to GTIE ranked at the bottom. The construction of China’s venture capital system is not perfect, the relevant laws and regulations are not perfect, and the fiscal and tax incentives are not enough. The positive impact of venture capital on GTIE needs to be further improved.

## 6. Conclusions and Policy Recommendations

This paper firstly constructed an improved super-SBM-DEA efficiency measurement model and combined the window analysis method to measure the GTIE of Chinese provinces from 2006 to 2018. Then, based on the PVAR model, the impulse response function was used to study the dynamic impact of technology and finance on the GTIE of China’s provinces. Finally, the variance decomposition was used to explore the contribution degree of variables of technology and finance: government financial technology investment, enterprise independent R&D investment, bank technology credit, and venture capital to improving GTIE. The main conclusions are as follows:

From 2006 to 2018, the average value of GTIE in China’s provinces was 0.42, which was at a relatively low level. The average GTIE increased from 0.34 in 2006 to 0.55 in 2018, showing a fluctuating upward trend during the research period. Impulse response results showed that government financial technology investment, enterprise independent R&D investment, bank technology credit, and venture capital investment had a positive impact on the GTIE, and the impact degree first increased and then decreased during the study period. The variance decomposition results showed that the GTIE had a high degree of autocorrelation, indicating that improving the GTIE was not achieved overnight but formed through long-term development and accumulation. Government financial technology investment contributed the most to the GTIE, followed by bank technology credit. The contribution degree of enterprise independent R&D investment of ranked third, and the contribution degree of venture capital to GTIE was the lowest. Based on the above research conclusions, policy recommendations were put forward which are shown in Table 6.

## Figures and Tables

**Table 1:** Descriptive statistics.

Indicator Type	Indicator	Mean	Standard Deviation	Maximum	Minimum
Innovation input	Full-time equivalent of R&D personnel (10,000 people/year)	10.117	11.605	76.273	0.121
	R&D capital stock (100 million yuan)	972.033	1276.673	7142.372	6.062
Expected output	Technology market turnover (100 million yuan)	227.513	542.250	4957.825	0.535
	New product sales revenue (100 million yuan)	3741.958	5732.126	39,376.056	8.566
Unexpected output	Industrial wastewater discharge (100 million tons)	7.246	6.080	28.718	0.578
	Industrial waste gas emissions (100 million standard cubic meters)	19,556.133	15,753.731	91,256.203	860.000
Technology and finance	Government financial technology investment (*gov*)	0.240	0.131	0.608	0.069
	Enterprise independent R&D investment (*com*)	0.706	0.141	0.914	0.329
	Bank technology credit (*fin*)	0.038	0.022	0.157	0.005
	Venture capital investment (*vc*)	0.085	0.213	2.879	0.000

**Table 2:** The measurement results of GTIE.

Province/Year	2006	2007	2008	2009	2010	2011	2012	2013	2014	2015	2016	2017	2018	Mean
Beijing	0.61	0.88	1.07	0.82	0.90	1.05	1.01	1.02	1.05	0.92	1.03	1.02	1.03	0.95
Tianjin	0.78	0.83	1.02	0.93	0.93	0.92	0.90	1.04	0.96	0.98	1.01	0.66	0.67	0.89
Hebei	0.14	0.17	0.19	0.17	0.17	0.20	0.24	0.19	0.16	0.17	0.20	0.27	0.49	0.21
Shanxi	0.12	0.17	0.19	0.17	0.14	0.19	0.21	0.25	0.21	0.20	0.22	0.34	0.47	0.22
Inner Mongolia	0.30	0.29	0.26	0.23	0.30	0.23	0.44	0.23	0.14	0.14	0.09	0.15	0.15	0.23
Liaoning	0.27	0.29	0.31	0.36	0.30	0.37	0.41	0.43	0.41	0.39	0.39	0.43	0.50	0.37
Jilin	0.37	0.35	0.41	1.08	0.32	0.46	0.37	0.18	0.28	0.26	0.63	0.80	0.65	0.47
Heilongjiang	0.10	0.18	0.18	0.18	0.16	0.15	0.19	0.19	0.19	0.19	0.17	0.23	0.25	0.18
Shanghai	1.00	1.01	1.03	0.94	0.92	0.96	0.78	0.69	0.76	0.63	0.79	1.02	1.01	0.89
Jiangsu	0.24	0.32	0.33	0.28	0.37	0.49	0.53	0.56	0.57	0.51	0.52	0.52	0.54	0.44
Zhejiang	0.29	0.30	0.31	0.23	0.21	0.30	0.54	0.84	0.62	0.54	1.01	0.66	0.79	0.51
Anhui	0.23	0.27	0.27	0.28	0.35	0.45	0.46	0.51	0.57	0.55	0.61	0.69	0.72	0.46
Fujian	0.30	0.31	0.30	0.27	0.29	0.29	0.30	0.24	0.18	0.20	0.16	0.22	0.21	0.25
Jiangxi	0.19	0.22	0.20	0.15	0.23	0.26	0.34	0.39	0.41	0.44	0.57	0.61	0.58	0.35
Shandong	0.21	0.31	0.33	0.32	0.34	0.34	0.33	0.35	0.39	0.37	0.41	0.48	0.46	0.36
Henan	0.19	0.23	0.22	0.21	0.18	0.22	0.19	0.22	0.20	0.20	0.20	0.24	0.35	0.22
Hubei	0.21	0.29	0.35	0.29	0.31	0.34	0.42	0.59	0.67	1.01	0.82	0.93	1.01	0.56
Hunan	0.39	0.39	0.40	0.39	0.36	0.36	0.59	0.74	0.64	1.01	0.69	0.80	0.64	0.57
Guangdong	0.44	0.39	0.50	0.41	0.73	0.58	0.67	0.58	0.50	1.03	0.83	1.03	1.03	0.67
Guangxi	0.09	0.09	0.18	0.11	0.16	0.18	0.07	0.18	0.18	0.15	0.39	0.46	0.44	0.21
Hainan	1.11	0.62	0.64	0.06	0.35	0.39	0.11	0.32	0.07	0.15	0.17	0.17	0.16	0.33
Chongqing	1.04	0.68	1.08	0.61	1.06	1.07	0.63	0.69	1.04	1.01	1.04	1.03	0.56	0.89
Sichuan	0.16	0.18	0.23	0.25	0.18	0.24	0.24	0.28	0.30	0.33	0.31	0.35	0.50	0.27
Guizhou	0.07	0.07	0.15	0.12	0.23	0.31	0.23	0.23	0.24	0.23	0.25	0.35	0.46	0.23
Yunnan	0.18	0.28	0.17	0.16	0.13	0.17	0.29	0.25	0.26	0.22	0.22	0.26	0.25	0.22
Shaanxi	0.09	0.12	0.14	0.18	0.21	0.31	0.35	0.45	0.47	0.47	0.47	0.53	0.61	0.34
Gansu	0.28	0.32	0.31	0.30	0.34	0.40	0.46	0.52	0.55	0.49	0.39	0.41	0.41	0.40
Qinghai	0.28	0.43	0.58	0.45	0.29	0.48	0.20	0.32	0.20	0.71	0.86	0.90	1.10	0.52
Ningxia	0.11	0.11	0.14	0.13	0.12	0.22	0.23	0.15	0.18	0.19	0.14	0.22	0.33	0.17
Xinjiang	0.27	0.26	0.30	0.09	0.21	0.18	0.18	0.12	0.11	0.09	0.09	0.09	0.05	0.16
Mean	0.34	0.35	0.39	0.34	0.36	0.40	0.40	0.43	0.42	0.46	0.49	0.53	0.55	0.42

**Table 3:** Panel unit root test results.

Testing Method	*te*	*gov*	*com*	*fin*	*vc*
LLC	−10.255 ***	−6.012 ***	−6.868 ***	−7.106 ***	−7.267 ***
ADF-Fisher	149.451 ***	100.709 ***	112.543 ***	135.519 ***	111.990 ***
Stability	Stable	Stable	Stable	Stable	Stable

Note: *** indicates significant at the level of 1%. The system standard errors are in parentheses.

**Table 4:** Impulse response results of GTIE faced with the shock of technology and finance.

Variable	Response Direction	Response Strength (Peak)	Response Speed	Cumulative Effect
*gov*→*te*	positive	0.0466	3	0.2428
*com*→*te*	positive	0.0208	2	0.0856
*fin*→*te*	positive	0.0177	3	0.0901
*vc*→*te*	positive	0.0061	2	0.0323

**Table 5:** Variance decomposition results of GTIE.

Response Variable	Pulse Variable	10th Period	15th Period	20th Period	25th Period	30th Period
*te*	*te*	0.642	0.626	0.623	0.622	0.622
	*gov*	0.281	0.297	0.300	0.301	0.301
	*com*	0.033	0.032	0.032	0.032	0.032
	*fin*	0.039	0.040	0.040	0.040	0.040
	*vc*	0.005	0.005	0.005	0.005	0.005

**Table 6:** Proposed policies for each indicator of technology and finance.

Indicators	Proposed Policies
Government	The government should increase financial investment in science and technology, optimize its structure, and ensure that financial investment in science and technology is mainly used to support basic research, cutting-edge technology research, major common key technology research, and innovation environment construction for which the market mechanism cannot effectively allocate resources. They should pay more attention to long-term benefits and the green and sustainable development of the economy, reflect the forward-looking and overall nature of financial science and technology appropriations, give full attention to the guiding role of policy funds, and guide more social capital into the field of technological innovation. The government should give full attention to the top-level design and guidance functions of technology and finance, make up for market failures in technology and finance, strengthen overall coordination, and choose the focus of support according to the laws and characteristics of innovation, the growth stage of science and technology enterprises, and the laws of industrial development. The government can build a comprehensive information sharing platform for science and technology enterprises to solve the problem of information asymmetry, and make the information of enterprises’ various resource advantages, production and operation, patent application authorization, bank-enterprise cooperation, and credit record open and transparent, which can help banks improve the screening ability and credit support for science and technology enterprises.
Enterprise	Enterprises are powerful carriers to realize technological innovation, and the best practitioners to lead high-quality economic development through technological innovation. Enterprises should increase the proportion of R&D investment in their own funds, and devote themselves to the research and transformation of cutting-edge technologies, key technologies, common technologies, and green and sustainable technologies. They should improve the independent innovation ability of enterprises, and make enterprises truly become the main body of research and development investment, technological innovation activities, and the application of innovation results. It is necessary to speed up the improvement of laws and regulations on the protection of independent intellectual property rights, establish an effective incentive mechanism, create a policy environment that encourages independent innovation of enterprises, stimulate the vitality of independent innovation of enterprises, affirm the spirit of entrepreneurship, and enable enterprises to become the main force in improving the GTIE. It is necessary to give full support to the important role of leading large enterprises in core technological innovation, build an open technological innovation system, allocate technology and finance resources to core technological industries, and cultivate a group of high-tech leading enterprises with world influence. At the same time, it is necessary to build a long-term mechanism to support the survival and development of small and medium-sized enterprises, and provide more inclusive financial services for them.
Bank	We must guide banks to give preferential credit support to national major science and technology achievements transformation projects and high-tech industrialization projects. Banks should launch financial products suitable for various development stages of science and technology enterprises and actively carry out investment-loan linkage business. The combination of bank technology credit and equity investment can not only diversify the credit risk of banks but also enhance the availability of loans for science and technology enterprises. We must encourage banks to develop new technology loan businesses such as intellectual property mortgages and collective entrusted loans, improve the guaranteed loan system that encourages enterprises to innovate, and reduce bank technology credit risks through cooperation between commercial banks and guarantee institutions, improve the bank’s technology credit system, build an evaluation system for small- and medium-sized enterprises that are suitable for their innovative development, and focus on solving the development problems of difficult and expensive financing for small- and medium-sized technology-based enterprises. Banks should comprehensively supervise the issuance of technology credits, improve their assessment capabilities before loan projects are issued, and their post-loan tracking and risk management capabilities, so as to ensure the efficiency of the use of funds.
Venture Capital	At present, there is still a lot of room for venture capital investment to improve the GTIE in Chinese provinces. To guide venture capital institutions to grow and develop, the government still needs to do further work in terms of tax incentives, investment environment improvement, and investment direction guidance. We must establish a risk compensation fund to appropriately subsidize the losses incurred by venture capital institutions in high-tech projects. Venture capital institutions should actively participate in the management and operation of the projects they invest in, and achieve mutual benefit and win-win results between venture capital institutions and innovative enterprises through long-term investment and common development. The capital investment of venture capital institutions should be inclined towards the R&D innovation process and the transformation of achievements of science and technology enterprises, which not only improves the technological innovation ability of science and technology enterprises but also enhances the commercial value of science and technology achievements, which is conducive to stimulating the investment enthusiasm of venture capital institutions. Venture capital institutions should focus on the new economy and new business forms generated by the integration of industrial Internet, artificial intelligence, big data, cloud computing, digital technology, and the real economy, and improve the efficiency of the use of technology and finance resources.

## Data Availability

Not applicable.
